# Spontaneous Ptyalism

**Published:** 1870-07

**Authors:** C. W. Knight

**Affiliations:** U. S. A. of Post Lampasas, Texas.


### ARTICLE IX.

### Spontaneous Ptyalism.

By C. W. Knight, M. D., U. S. A.

Of Post Lampasas, Texas.

On board the U.S. steamer "New England," at Havana, on the 3d February last. Mr. I. L. made me the following statement: Two days previously he had noticed that his mouth was unusually "watery;" but he had not thought much of it until waking this morning he found his pillow and night-shirt saturated with saliva. He also said that he felt a "little stiff and sore in the jaws." He stated positively —and I place great confidene in his desire to tell me the entire truth—*that he had been taking no medicine of any kind*, with the exception of two Seidlitz powders, which I had given him to relieve a tendency to constipation. On examination I found the parotids slightly enlarged and indurated with increased sensibility to touch. The gums were swollen and moderately sensitive around the molars. The tongue looked healthy, with the exception of a little whitish fur near the base. The breath was offensive, resembling in odor that of the body of an uncleanly man. On careful inspection the teeth were found to be perfectly sound, and entirely free from any sharp edges that might act as sources of irritation. Being his messmate, I knew that his diet had contained a full allowance of fresh meats and vegetables. I had many opportunities for knowing that his health had been good in every respect, excepting that his bowels had been constipated, as previously mentioned.

As treatment, I ordered a purge of rhubarb and jalap to be taken at once. Internally, a tablespoonful, every three hours, of a solution of potassæ chlor. of the strength of one drachm to the pint; this solution to be also used as a mouth wash several times daily.

*Feb. 4th*—All the symptoms much aggravated. Parotids larger and more painful; gums hot, tense, and very sensitive; tongue coated; yellowish in the centre, whitish on the edges and tip, with indications of forming ulcers on the edges. Patient very nervous and irritable, and complaining much of pain at the angles of the jaw. Marked fetor of breath. ℞. Tinct. ferri subsulph., gtt. v every three hours; pulvis. ipecac. comp. gr. x, twice in the day; continue the internal and local application of the potash solution, but of doubled strength

*Feb.* 5*th.*— Worse. Tongue much swollen, with ulcers in several places on its edges; between three and four pints of saliva poured out during the night; parotid and submaxillary glands, enlarged, hard, and painful. The patient's appearance is pitiable, and reminds one of the description by old writers of severe cases of mercurial salivation. Ordered a nourishing diet, with a milk punch, twice a day. Continued tinct. ferri subsulph., gtt. x, every three hours, and potash solution; Dover's powder *pro re nata.*

*Feb.* 6*th.*—No change for the better. The ulcers on the tongue have run together forming three large sores; an ulcer on each cheek as large as a silver dollar, with rugged edges, and of adark reddish color; these ulcers present a very unhealthy apperance; ordered beef essence, with milk punch; continue iron, omit chlorate of potash, and subsitute a saturated solution of chloride of sodium, with which the mouth is to be thoroughly washed *every half hour;* the ulcers on the tongue and cheeks being touched, in addition, three or four times during the day with a crystal of rock salt.

*Feb.* 7*th.*—From the commencement of the chloride of sodium treatmeut the patieat improved rapidly, the frequency of application being gradually decreased as the patient grew better. The tonic regimen was kept up for ten days. He was entirely well in two weeks.

I desire particularly to call attention to the great relief experienced from the use of the chloride. At every application the patient said a cool and pleasant sensation was imparted to his mouth. He soon acquired the habit of keeping a small lump of salt in the mouth, and said it was as pleasant as a piece of ice to his parched tongue. The fetor of his breath was markedly controlled by the salt, so that the almost unbearable odor of the cabin was soon dissipated.

I am unable to assign any cause for the complaint, unless the torpidity of his bowels can be charged with it. I have called it spontaneous ptyalism after Thomas Watson, who gives, in his "Practice of Physic," that name to a similar train of symptoms. The mode of invasion and the severity of the symptoms distinguished my case, I think, from mumps, or ordinary stomatitis.—*Med. and Sur. Reporter.*

